# Comparison of General Anesthesia (Sevoflurane) and Spinal Anesthesia (Levobupivacaine) Methods on QT Dispersion in Inguinal Hernia Operations

**DOI:** 10.7759/cureus.9079

**Published:** 2020-07-08

**Authors:** Basak Pehlivan, Murat Akçay, Ahmet Atlas, Mehmet K Erol, Erdogan Duran, Mahmut A Karahan, Orhan Binici, Evren Büyükfırat, Nuray Altay

**Affiliations:** 1 Anesthesiology, Harran University, Sanliurfa, TUR; 2 Anesthesiology and Reanimation, Ankara Numune Training and Research Hospital, Anesthesiology and Reanimation Clinic, Ankara, TUR; 3 Anesthesiology and Reanimation, Harran University, Sanliurfa, TUR; 4 Anesthesiology and Critical Care, Harran University, Sanliurfa, TUR

**Keywords:** sevoflurane, levobupivacaine, qt interval, arrhythmia

## Abstract

Introduction

Arrhythmias are one of the most frequently seen issues during surgical operations. In this study, we investigated and compared the effects on the QT dispersion of patients when using a method of volatile inhalation mask anesthesia with sevoflurane (VIMA: Group I) and when spinal anesthesia was performed with levobupivacaine (Group II).

Methods

The study included 40 patients who had American Society of Anesthesiology scores of I-II (ASA I-II), were aged from 18 to 65 years, and were scheduled for inguinal hernia operations. Approval of the university ethics committee was obtained before the study began. All patients had measurements taken for non-invasive blood pressure, including systolic arterial pressure (SAP), diastolic arterial pressure (DAP), and mean arterial pressure (MAP), heart rate (HR), and oxygen saturation (SO_2_) values. The QT intervals were measured using the 12-derivation electrocardiogram (ECG) device (Cardiofax V). Our study was performed with randomization using the closed envelope method.

Results

When the percentage differences of the HR values from the initial period in both groups were compared, we observed significant differences between the groups, with increases in the VIMA group at the second period as well as increases in the VIMA group at the fourth, fifth, sixth, seventh, and ninth periods but decreases in the spinal anesthesia group for these periods. There were statistically significant differences between the two groups at the third and fifth periods when the percentage differences of the QTc values from the initial period were compared. We observed increases in the spinal anesthesia group.

Conclusion

In our study, we suggest that the tendency toward arrhythmia may be reduced by choosing general anesthesia with sevoflurane rather than levobupivacaine in patients with cardiac complaints who are undergoing regional anesthesia and/or taking medication that affects QT intervals.

## Introduction

One of the most frequent problems that anesthetists see during the perioperative period is arrhythmias. It is important for anesthetists to identify the causes of arrhythmias during this period, and there may be many reasons for such arrhythmias. Among the causes that are considered are anesthetic agents, local anesthetic agents used for regional anesthesia, abnormal arterial blood gas and electrolyte values, endotracheal intubation and similar incidents causing catecholamine release, existing heart disease, surgical manipulation, interventions performed on patients, and other medications the patients use. Many studies suggest that altered blood electrolyte levels, hypoxia, and acidosis also contribute to the occurrence of arrhythmia [1].

The differences between the longest QT intervals and the shortest QT intervals on the electrocardiograms (ECGs) are referred to as QT dispersion (QTd), while this is called corrected QT dispersion (QTcd) if corrected QT intervals are used. These show the regional differences of the QT intervals. Values between 40 ms and 50 ms are considered to be normal. This is also considered as an indicator of left ventricular repolarization homogeneity, which may represent an electrophysiological index for the risk of ventricular dysrhythmia [2]. Increases in QTd indicate heterogenous repolarization and possible arrhythmias [3]. The inter-derivational differences of the QT intervals indicate differences in regional repolarization. The homogeneity of ventricular repolarization decreases as much as the QTd increases and also, therefore, means increased ventricular instability [4].

It is already known that some local anesthetics may cause ventricular arrhythmia through a re-entry mechanism. QTd causes ventricular dysrhythmia (especially polymorphic ventricular tachycardia). General and spinal methods of anesthesia are those that are most frequently used in inguinal hernia operations. Central catheterizations, peritoneum traction, tracheal traction, and compression on the eyes and brain may cause arrhythmia [1-5]. General anesthesia and spinal anesthesia may also cause these dysrhythmias.

In this study, we aimed to investigate the effects of volatile inhalation mask anesthesia (VIMA) with sevoflurane and spinal anesthesia with levobupivacaine, which are both well-known to have potential for cardiac arrhythmia, in relation to the QTd of patients in various age and gender groups when used in inguinal hernia operations.

## Materials and methods

The study included 40 patients, aged from 18 to 65, who were scheduled for inguinal hernia operations and had American Society of Anesthesiology risk scores of I-II (ASA I-II). Approval was obtained from the ethical committee of Harran University before the study began. Patients were informed in the preoperative period about the types of anesthesia that would be used, and their consents were obtained. No preoperative premedication drug was given. Our study was randomized using the closed envelope method.

Patients were divided into two groups: Group I was the VIMA (sevoflurane) group (n:20); Group II was the spinal anesthesia (levobupivacaine) group (n:20).

Patients with pre-existing cardiac disease, arrhythmia that was identified on the ECG, use of medications that affects the QT interval, electrolyte imbalance, alcohol and smoking habits, diabetes mellitus, abnormal biochemical and hemogram values, and possible difficulty with intubation were excluded from the study.

Both groups were monitored according to standard practice. The parameters that were monitored included non-invasive blood pressure, systolic arterial pressure (SAP), diastolic arterial pressure (DAP), mean arterial pressure (MAP), heart rate (HR), and oxygen saturation (SO_2_). The measurements were taken in a total of nine periods including the initiation period. Measurements of the QT intervals were obtained through 12-lead ECGs (Cardiofax V ECG device). A total of six periods were taken, including the QT intervals measurements initiation period.

All ECGs were obtained as standard 12-lead with 25 mm/sec paper rates. In all derivations of each ECG, the starting points that the QRS complex separates from the isoelectric line and the returning point of the T wave to the isoelectric line were identified. These were identified separately and measured by a cardiologist who was unaware of the clinical situation. If there was a U wave, the lowest point of the merging zone of the T and U waves was considered to be the end of the T wave. If the exact end point of a T wave could not be detected, it was removed from the measurement. Patients who were measured with at least seven derivations (which included at least three chest derivations) were included in the study.

The “Bazett formula” was used for correction of the QT interval according to rates [6].

QTd = QTmax - QTmin

QTc = QT / RRı

QTcd = QTcmax - QTcmin

For Group I, induction by tidal volume technique with 8% sevoflurane in O2/N2O (50%/50%) was initiated after monitorization. The sevoflurane ratio was then gradually decreased to 2-3%, and 0.6 mg/kg rocuronium was administered through intravenous (IV) at the third minute of induction. Patients were intubated by the same person at three minutes after receiving muscle relaxants. Anesthesia maintenance was sustained with 2-3% sevoflurane. After each surgery was finished, the N2O and sevoflurane were stopped, and 100% O2 was given. In the sevoflurane group, no anticholinergic agent was administered to any patient for residual effects of the muscle relaxants.

For Group II, 15 mg/kg Ringer lactate infusions were administered to the patients before their procedures. Patients were properly positioned (sitting position) after monitorization. Clearance and coverage were performed according to the rules for sterilization and asepsis. The subarachnoid spaces were entered with sharp-edged spinal needles at the level of lumbar (L)3-4 and L4-5 areas, and 3 mL (15 mg) 0.5% isobaric levobupivacaine was administered for at least 15 seconds to patients after observing their cerebrospinal fluid (CSF) flows. The patients' positions were corrected, and the spinal blocks were evaluated every five minutes. The surgical procedures were started after adequate spinal levels had been obtained, and 3 L/min O2 was given to patients through masks during the operations (Tables 1, 2).

**Table 1:** Measurement periods of systolic arterial pressure (SAP), diastolic arterial pressure (DAP), mean arterial pressure (MAP), SpO2 and heart rate (HR) in Group I (sevoflurane VIMA) and Group II (levobupivacaine) during spinal anesthesia.

Period	Group I	Group II
Initiation period (IP)	0 min	0 min
1st period	VIMA 1st min	Spinal anesthesia 1st min
2nd period	VIMA 5th min	Spinal anesthesia 5th min
3rd period	VIMA 10th min	Spinal anesthesia 10th min
4th period	Incision 1st min	Incision 1st min
5th period	Incision 5th min	Incision 5th min
6th period	Incision 10th min	Incision 10th min
7th period	End of surgery	End of surgery
8th period	Postoperative 1st hour	Postoperative 1st hour

**Table 2:** Periods of ECG obtaining QTc, QTd and QTcd measurement in VIMA and levobupivacaine groups during spinal anesthesia.

Period	Group I	Group II
Initiation period (IP)	0 min	0 min
Period I	VIMA 5th min	Spinal anesthesia 1st min
Period II	Intubation 1st min	Spinal anesthesia 10th min
Period III	Surgical incision 1st min	Surgical incision 1st min
Period IV	End of surgery	End of surgery
Period V	Postoperative 1st hour	Postoperative 1st hour

Statistical method

The SPSS 24 (SPSS® for Windows, IBM Corp., Armonk, NY) software program was used for statistical analysis. Numeric data were presented as means ± standard deviations. The Kolmogorov-Smirnov test was performed for evaluating distribution of numeric data. The independent samples t-test was used when the distribution of the numeric data was normal, whereas the Mann-Whitney U test was used when it was abnormal. The one-way analysis of variance (ANOVA) test was used for inter-group comparisons when the distribution of numeric data was normal. The Bonferroni test was used as a post hoc test. In addition, the Kruskal-Wallis H test was used for comparison when the distribution was abnormal, whereas the Mann-Whitney U test was used for paired comparison if the results were significant. The Chi-square test was used for the comparison of non-numeric data. Results with a p-value < 0.05 were considered statistically significant.

## Results

In evaluating the demographics of the participants, there were no statistically significant differences in terms of age, weight, and length (p > 0.05) between the two groups. There were also no statistically significant differences between the groups in terms of SpO2 during perioperative monitoring (p > 0.05). The patients also received ephedrine when hypotension occurred after the blocks. Although ephedrine also affects QTc, it was not included in the evaluation because the participants in both groups of the study received similar ephedrine dosages [7].

**Figure 1:**
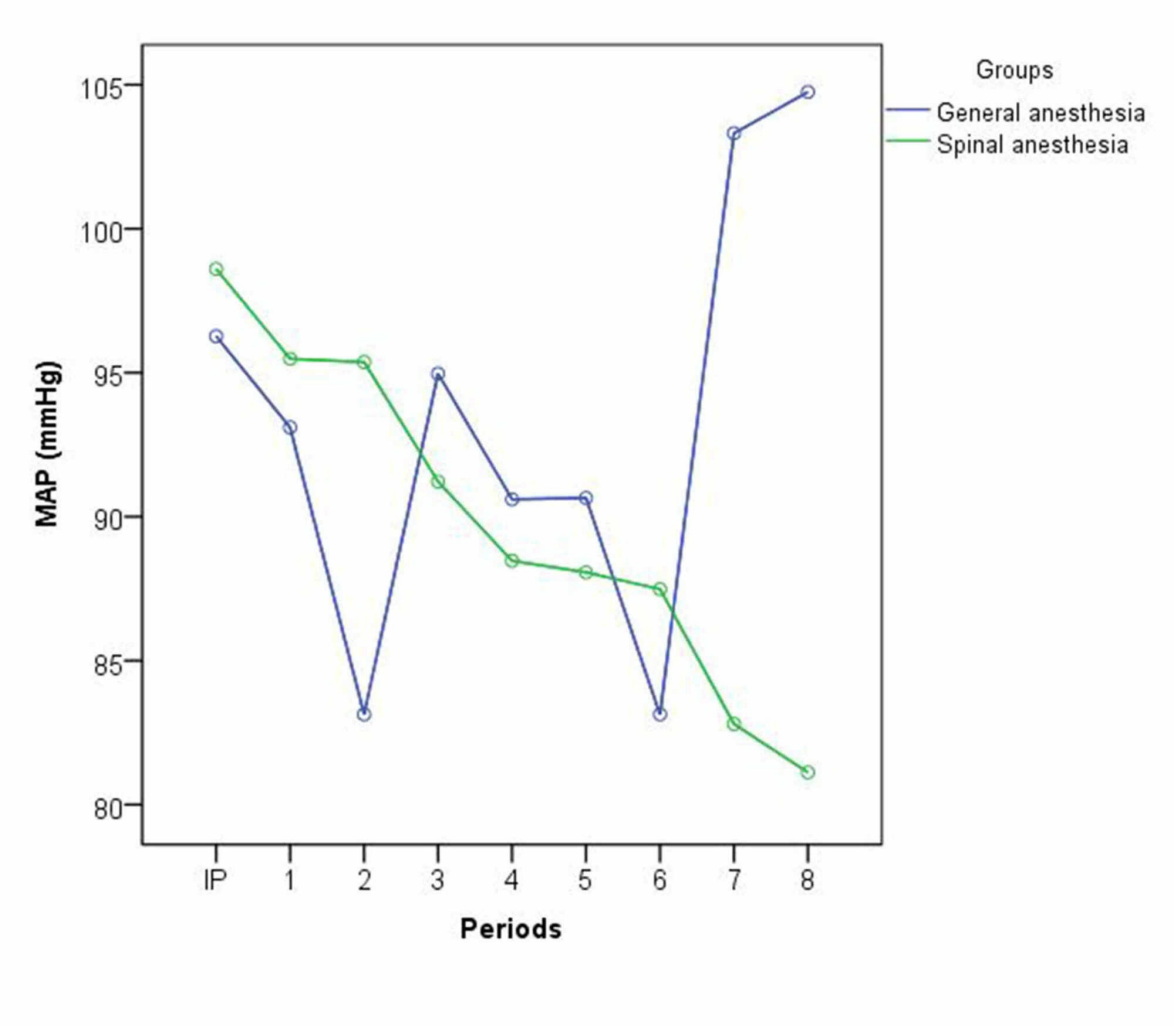
Mean arterial pressure comparison in Group 1 (VIMA = sevoflurane) and Group 2 (Levobupivakaine). MAP: Mean arterial pressure; IP: Initiation period; VIMA: Volatile inhalation mask anesthesia.

When the percentage changes of the SAP, DAP, and MAP values from the initiation periods in both groups were evaluated, it was found that there were statistically significant differences between the decreases in the third period for Group I, the increases in the seventh and eighth periods for Group I, and the decreases in those periods for Group II (p < 0.05) (Figure 1).​​​​​​

When percentage changes of HR from initiation period in both groups were compared, it was found that there was a significant difference between increase in 2nd period of Group I, and 4th, 5th, 6th, 7th and 8th periods of Group I and decrease in Group II (p < 0.05).

**Figure 2:**
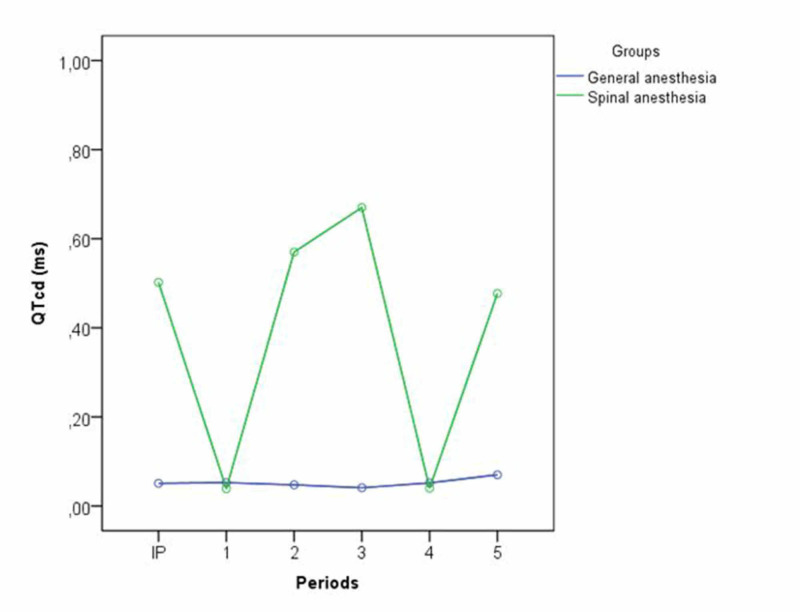
QTcd (ms) comparison in Group 1 (VIMA = sevoflurane) and Group 2 (Levobupivakaine). IP: Initiation period; VIMA: Volatile inhalation mask anesthesia.

When the percentage changes of the QTd and QTcd values from the initiation periods in both groups were compared, the differences in the increases in Group II and those in Group I at the third, fourth, and fifth periods were statistically significant (p < 0.05) (Figure 2).

## Discussion

Our study evaluated the effects of general anesthesia performed with sevoflurane and spinal anesthesia with levobupivacaine on QT dispersions in inguinal hernia operations. According to the results, we demonstrated more prolonged QTc, QTd, and QTcd intervals with levobupivacaine than with general anesthesia using sevoflurane.

The mechanism with regard to arrhythmias in patients with predisposing factors is well-defined. However, there is still insufficient information about the causes of arrhythmias that are seen in the perioperative period in patients who do not have predisposing factors. Drugs for local anesthesia have potential for cardiovascular toxicity because they do not only block the ion channels in the nerve cell membranes but also those in excitable tissues such as the heart [8, 9]. One study proposed that alterations in the use of intracellular Ca2+, especially with impaired Ca2+ channels and sarcoplasm reticulum (SR) functions, may be responsible because of the adverse myocardial effects of left atrial appendage (LAA) in local anesthetic toxicity [10]. Also, the risk of toxicity is even greater in local anesthetics that have long-lasting effects [11].

Myocardial depression that is observed with amide type local anesthetics can also, in theory, be observed with levobupivacaine. However, the manufacturer of levobupivacaine has defined possible cardiotoxicity as a decrease in cardiac output, hypotension, and ECG changes showing a heart block, bradycardia, or ventricular tachyarrhythmias, which may cause cardiac arrest [12]. In our study, a comparison of the QTcd prolongation with levobupivacaine to that with sevoflurane, especially at the third, fourth, and fifth periods, was consistent with these studies [13,14].

It is known that volatile anesthetics such as sevoflurane significantly prolong the QT intervals [15]. In addition, Yildirim et al. reported that sevoflurane, isoflurane, and desflurane prolong the QTc and QTcd intervals, and Silay et al. found that sevoflurane and desflurane prolong the QTc intervals but do not affect the QTd intervals [16,17].

Another study found that sympathetic stimulation during laryngoscopy and intubation may worsen QTc interval prolongation, and QTc interval prolongation that is partially prevented due to weakened sympathetic stimulation during intubation was possibly due to premedication with fentanyl [18]. In our study, no agent was given during intubation or extubation for the sevoflurane group. However, there was no effect on the QT, although the MAP and HR increased.

A study by Terao et al. reported no prolongation in the QTc intervals during anesthesia induction with propofol and sevoflurane. Therefore, they suggested that propofol and sevoflurane are appropriate for anesthesia induction in patients with risk factors for ventricular arrhythmia [19]. Akçay et al. linked these increases to the sympathetic discharge during intubation and extubation [20]. This study was consistent with our study, which found MAP and HR increases in Group I during the intubation and extubation periods. However, the QT was prolonged when compared to levobupivacaine.

Prolongation of drug-related repolarization may induce malignant ventricular arrhythmias such as torsades de pointes (TdP). It has been reported that all inhalation anesthetics, especially isoflurane and desflurane, prolong QTc and QTcd, but sevoflurane probably has no effect on the transmural dispersion of repolarization (TDR) [21]. In addition, reversion of neuromuscular block by anticholinesterase-anticholinergic combinations is related to important QTc prolongation. Anticholinesterase-anticholinergic agents were not used in our study (especially in the sevoflurane group), although we tried to demonstrate the effect of sevoflurane on QTc. In our study, the effect of sevoflurane on QTc was compatible with this study [21].

In their daily routines, anesthetists observe secondary prolongation of cardiac repolarization relatively frequently, because many anesthetic drugs are used to prolong QTc intervals. Prolongation rarely causes serious outcomes; however, it may cause TdP and ventricular fibrillation [22]. Sevoflurane is considered to be a safe anesthetic in long QT syndrome (LQTS) patients [22,23].

Central neuraxial blocks may affect cardiac electrophysiology because of induced autonomic imbalance. Indeed, subarachnoid blocks among regional modalities are probably the most prevalent cardiovascular effects and are related to the block rate, power, and degree of the sympathetic blockage [24]. Owczuk et al. reported that significant QTc prolongation started one minute after a subarachnoid block [22, 24]. In our study, QTd and QTcd prolongations at the third, fourth, and sixth periods in the spinal anesthesia group that received levobupivacaine were consistent with findings by Owczuk et al.. Their study demonstrated that the QTc and T wave (Tp-e) interval were not affected by intrathecal 10-15 mg bupivacaine administration in patients undergoing cesarean delivery [25,26]. According to the results of our study, sevoflurane that is used in general anesthesia is safer than levobupivacaine that is used in regional anesthesia.

Gristwood and Bardsley administered 0.5% levobupivacaine and bupivacaine intravenously in human volunteers and found a mild increase in corrected QT (QTc) intervals in both drugs [8,27,28]. In a study by Dogan et al., at the end of the operation, the prolongation of the QTc interval disappeared in the bupivacaine group, but continued in the levobupivacaine group, then disappeared [29]. Again in this study, levobupivacaine was compatible with our group.

## Conclusions

Arrhythmias are perhaps the most important of the problems encountered by anesthesiologists in the perioperative period. Determining the cause of arrhythmias in the perioperative period is important for the anesthetist. Many causes can cause arrhythmia in the perioperative period. In conclusion, we believe that the tendency toward arrhythmia may be reduced by using general anesthesia with sevoflurane rather than levobupivacaine in patients who have cardiac complaints and/or are taking medications that affect QT and who can also undergo regional anesthesia.
